# Psychological Distress in Intracranial Neoplasia: A Comparison of Patients With Benign and Malignant Brain Tumours

**DOI:** 10.3389/fpsyg.2021.664235

**Published:** 2021-08-16

**Authors:** Michael Karl Fehrenbach, Hannah Brock, Anja Mehnert-Theuerkauf, Jürgen Meixensberger

**Affiliations:** ^1^Department of Neurosurgery, University Medical Center Leipzig, Leipzig, Germany; ^2^Department of Medical Psychology and Medical Sociology, University Medical Center Leipzig, Leipzig, Germany

**Keywords:** intracranial neoplasia, psychological distress, self-report questionnaires, depression, anxiety, posttraumatic stress disorder, health-related quality of life, fear of progression

## Abstract

**Objective:** We aimed to assess psychological distress in patients with intracranial neoplasia, a group of patients who suffer from severe functional, neurocognitive and neuropsychological side effects, resulting in high emotional distress.

**Methods:** We conducted a cross-sectional study, including inpatients with brain tumours. Eligible patients completed validated self-report questionnaires measuring depression, anxiety, distress, symptoms of posttraumatic stress disorder (PTSD), fear of progression and health-related quality of life. The questionnaire set was completed after brain surgery and receiving diagnosis and before discharge from hospital.

**Results:** A total of *n* = 31 patients participated in this survey. Fourteen of them suffered from malignant (*n* = 3 metastatic neoplasia) and 17 from benign brain tumours. Mean values of the total sample regarding depression (*M* = 9.28, SD = 6.08) and anxiety (*M* = 6.00, SD = 4.98) remained below the cut-off ≥ 10. Mean psychosocial distress (*M* = 16.30, SD = 11.23, cut-off ≥ 14) and posttraumatic stress (*M* = 35.10, SD = 13.29, cut-off ≥ 32) exceeded the clinically relevant cut-off value in all the patients with intracranial tumours. Significantly, more patients with malignant (79%) than benign (29%) brain tumours reported PTSD symptoms (*p* = 0.006).

**Conclusion:** Distress and clinically relevant PTSD symptoms in patients with intracranial neoplasia should be routinely screened and treated in psycho-oncological interventions immediately after diagnosis. Especially, neuro-oncological patients with malignant brain tumours or metastases need targeted support to reduce their emotional burden.

## Introduction

The average annual age-adjusted incidence of primary brain and nervous system tumours among adults (≥ 40 years) is estimated to be 44.47 per 100,000 of the population. One-third of these are malignant CNS tumours, which is the eighth most common cancer among men and the fifth among women in this age group. Meningioma is the most common benign entity, whereas glioblastoma is the most common malignant intracranial tumour, except for metastases (Ostrom et al., 2018). It is noteworthy that 20–40% of all patients diagnosed with an invasive solid malignancy originating outside the CNS develop brain metastases during the progression of their disease (Cagney et al., 2017).

Neuro-oncologic therapy strategies range from observation for benign tumours to complex multimodal treatments for malignant entities. Typically, in glioblastoma, surgery is followed by chemo-radiotherapy and then the continuation of chemotherapy only. Disease progression often results in serious side effects, such as paralysis, epileptic seizures, aphasia or changes in personality.

The majority of patients with brain tumours suffer from anxiety and depressive mood states that commonly manifest as psychological distress (Randazzo and Peters, 2016). The National Comprehensive Cancer Network guidelines describe distress as a multifactorial unpleasant experience (e.g. a cancer diagnosis) that may interfere with coping skills, ranging from common feelings of sadness and fears to severe reactions that can be diagnosed as psychiatric illnesses (Riba et al., 2019). Recent studies have found that up to 48% of patients with malignant brain tumours exhibit signs of depression and anxiety and 56% report elevated distress during the hospital stay that remains stable over time (Ford et al., 2012; Singer et al., 2018; Goebel and Mehdorn, 2019). In line with this, patients with cerebral metastases suffer from similar morbidity and mortality and exhibit similar needs for supportive care as patients with non-metastatic malignant intracranial tumours (Maqbool et al., 2017).

Etiological factors can influence the interaction of cancer- and treatment-related, individual, and psychosocial factors that contribute to the risk of anxiety and/or depression. The following have been identified as risk factors for increased cancer-related distress: female gender, living alone, having children, lower income, longer duration of illness, younger age, a history of psychiatric disorders, substance use, physical/sexual abuse in the past or comorbid diseases (Randazzo and Peters, 2016). Patients with intracranial tumours experience higher emotional distress levels than patients with other cancer diagnoses (Carlson et al., 2004). They face a double burden of not only an oncological but also a neurological disease.

Given the invasive character of brain tumours, difficulties in coping with the disease, high unmet needs and the decreased health-related quality of life (HRQoL) are often prevalent (Halkett et al., 2015; Randazzo and Peters, 2016). This compilation can negatively impact patient adherence to continuing multimodal tumour treatments, which might adversely impact cancer therapy outcomes.

Seen against the background of these findings, the need for psycho-oncological care in these patients is high (Rosenberger et al., 2012; Renovanz et al., 2017). However, there is scant data available on the differences in distress and the emotional burden of patients with different brain tumour diagnoses (benign and malignant, and metastases). This is a topic worthy of investigation in the light of therapy intensity depending on the dignity (multimodal and intensive cancer therapy vs. surgery only). However, such assessments remain challenging as (i) most of the patients with brain tumours experience neurocognitive deficits and might not be able to complete questionnaires and (ii) many instruments have not been adapted to the diverse needs of neuro-oncological patients (Renovanz et al., 2020). There is a lack of neuro-oncologically evaluated tools, and currently, we are not aware of any screening tool that has been sufficiently tested in these patient groups. Also the European Association for Neuro-Oncology Guidelines recommended assessing the psychological support needs of patients with gliomas without naming specific diagnostic tools, presumably because corresponding instruments are hardly available (Weller et al., 2021).

Therefore, we conducted a single-centre study to investigate distress in patients with intracranial tumours after surgery and before discharge from hospital. We specifically aimed to explore (i) the occurrence of psychological distress, symptoms of depression and anxiety, fear of progression, posttraumatic stress symptomatology and HRQoL in patients with intracranial neoplasia and (ii) the differences in distress between patients with benign and malignant tumours.

## Materials and Methods

### Design and Procedure

Originally, this study was planned and conducted as a longitudinal study, including four measurement time points (t0–t3), but due to extremely high dropout rates (t1: 39%, t2: 53% and t3: 100%; mostly not due to death), we only analysed the data obtained at the first assessment (t0). So, we conducted a cross-sectional single-centre study that included patients who had been surgically treated for newly diagnosed intracranial neoplasia in the Department of Neurosurgery at the University Hospital Leipzig, Germany, between January 2017 and December 2017. The study protocol was approved by the Research Ethics Committee of the University of Leipzig, Germany (approval number 467/16-ck).

### Participants

Patients who met the following criteria were eligible for inclusion in this study: date of initial diagnosis during 2017, intracranial tumour with surgery as initial treatment, complete histological diagnosis and an interdisciplinary tumour board review to define therapy strategy before discharge from the hospital. We assume that only after the tumour board decision the differences in the distress patterns become apparent. Before the histologic results are announced, distress levels are elevated in all groups due to the expectation of a possibly poor prognosis. However, we were more interested in the inpatient distress level differences due to the therapy intensity depending on the dignity (multimodal cancer therapy vs. only surgery).

Patients were excluded from the study if they had a severe cognitive or functional deficit, brain abscess or CNS infection; advanced tumour disease with an estimated remaining life span of less than 6 months; were under the age of 18 years or were unable to complete the questionnaires in German. After written informed consent was obtained, sociodemographic data were collected.

Two patient groups were established according to tumour malignancy: benign tumours, for example, low-grade meningioma that requires no adjuvant tumour therapy (Group A) and malignant tumours, for example, glioblastoma or metastases that require combined chemo- and radiotherapy (Group B).

### Procedure

Patients were asked to complete the set of questionnaires after surgery and after being informed of their diagnosis, prognosis and required adjuvant therapy. Clinical assessment was routinely done on the day of discharge. The baseline data included gender, age, level of education, relationship status, children, epilepsy and tumour entity.

### Instruments

In contrast to previous studies, we used different instruments to gain a comprehensive and differentiated understanding of the distress. Although the questionnaires are well-established tools, there are only a minimal number, if any, concerning experience of neuro-oncologic diseases. However, to compensate for the lack of neuro-oncologically adjusted screening tools, we settled on a combination of seven questionnaires, which ultimately resulted in a long questionnaire containing a total of 78 items.

The Patient Health Questionnaire (PHQ-9) is the depression module of the PHQ-9, a self-administered version of the PRIME-MD diagnostic instrument for common mental disorders. It consists of nine items that are scored from ‘0’ (not at all) to ‘3’ (nearly every day; Kroenke et al., 2001). A PHQ-9 score of ≥ 10 has a sensitivity and specificity of between 83 and 95% for detecting major depression (Gräfe et al., 2004).

We used the Generalised Anxiety Disorder 7 (GAD-7) scale as a screening tool for symptoms of generalised anxiety disorder. It is a 7-item self-report scale with a maximum score of 21. The individual items range from ‘0’ (not at all) to ‘3’ (nearly every day). The GAD-7 scale scores can be divided into four levels: minimal (0–4), mild (5–9), moderate (10–14) and severe (15–21). A cut-off value of ≥ 10 provides a sensitivity of 89% and a specificity of 82% regarding anxiety (Spitzer et al., 2006; Esser et al., 2018).

In line with the PHQ-9, the 15-question somatic symptoms scale from the Patient Health Questionnaire (PHQ-15) is the somatic symptoms scale of the PHQ. It comprises 15 somatic symptoms, with each symptom scored from ‘0’ (not bothered at all) to ‘2’ (extremely bothered). Scores of 5, 10 and 15 represent good cut-off points for low, medium and high somatic symptom load (Kroenke et al., 2002; Gräfe et al., 2004).

The Questionnaire on Distress in Cancer Patients – short form (QSC-R10) is a self-assessment instrument concerning psychosocial distress in cancer patients. It is a 10-item questionnaire with a sensitivity of 81% and specificity of 73.2% that uses a cut-off score of >14 to detect distress (Herschbach et al., 2004; Book et al., 2011; Haun et al., 2014).

The SF-8 Health Survey is an alternate form of the SF-36 Health Survey, with only one question to measure each of the health domains. Each item has a 5- or 6-point response range. Lower values correspond to higher quality of life, whereas higher values are linked to higher discomfort. This instrument is used to measure HRQoL (Ware and Sherbourne, 1992; Turner-Bowker et al., 2003; Ellert et al., 2005).

The Posttraumatic Stress Disorder Checklist-Civilian Version (PCL-C) is a standardised self-report rating scale for PTSD, comprising 17 items that correspond to key symptoms of PTSD. A total symptom severity score is obtained by summing up each of the 17 items, which have response options ranging from ‘1’ (not at all) to ‘5’ (extremely). Higher values correspond to higher stress (Ruggiero et al., 2003). The optimal cut-off score proposed by a German validation study was ≥ 32 (Höcker and Mehnert, 2012).

The Fear of Progression Questionnaire short form (FoP-Q-SF; German version: PA-F-KF) is a questionnaire specifically developed for chronically ill patients. It is the shortened form of the much longer FoP-Q, with only 12 instead of 43 items. There are five possible answers per item, ranging from ‘1’ (never) to ‘5’ (very often). It has been tested and evaluated for neuro-oncologic patients. Higher scores are a good indication of increased fear of progression, which leads to significantly higher levels of stress. There is no universally applicable cut-off value, but responding to 50% or more of the questions with ‘often’ (4 points) or ‘very often’ (5 points) can be considered a good indicator of fear of progression (Herschbach et al., 2005; Mehnert et al., 2006, 2009).

### Statistical Analyses

We used IBM SPSS Statistics 25 for statistical data analyses. The means and standard deviations of sample characteristics, distress and symptom burden were calculated for the total sample, separated by dignity. Since our data did not follow a normal distribution, mean differences were tested with the nonparametric Mann-Whitney U-test. Furthermore, we used chi-square tests to analyse frequency differences. Regarding the sample size and to increase the usability of the data, we applied Yates correction for continuity. We used the mean square contingency coefficient phi to judge the magnitude of effects (Cohen, 2013). Effect sizes can be classified as small (*φ* ≥ 0.1), medium (*φ* ≥ 0.3) or high (*φ* ≥ 0.5).

## Results

### Sample Characteristics

**Figure 1:**
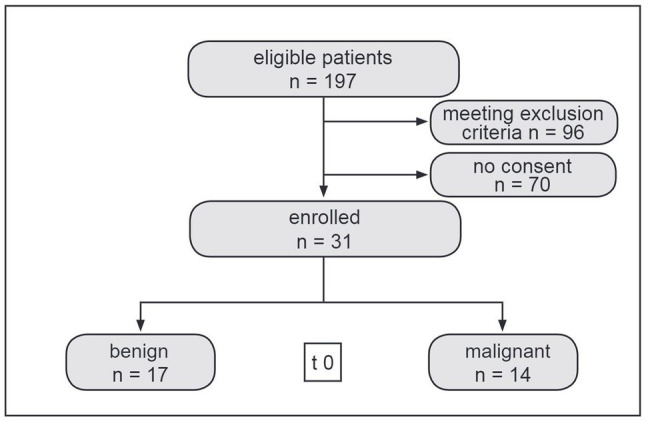
Flow chart depicting patient enrolment.

Out of 197 (79 females and 118 males) eligible patients with newly diagnosed intracranial tumours, 31 patients consented to participate in this study (Figure 1). Seventeen of them had been diagnosed with benign (Group A) and 14 with malignant (Group B) tumour entities (Table 1).

**Table 1:** Sample characteristics.

	All patients	Group A	Group B	*p*
	(*N* = 31) *n*(%)	(*n* = 17) *n*(%)	(*n* = 14) *n*(%)	
Gender				
Male	10(32.3)	6(35.3)	4(28.6)	0.990^1^^,^^2^
Female	21(67.7)	11(64.7)	10(71.4)	
Mean age (years)	61.32	59.71	63.29	0.340^3^
SD	17.14	17.33	17.34	
Range	19–86	19–86	27–80	
School education				
Compulsory	5(16.1)	1(5.9)	4(28.6)	
Post-compulsory	18(58.1)	12(70.6)	6(42.9)	0.170^1^
College	8(25.8)	4(23.5)	4(28.6)	
Relationship				
Partnership	24(77.4)	12(70.6)	12(85.7)	0.568^1^^,^^2^
Single	7(22.6)	5(29.4)	2(14.3)	
Children				
Yes	22(71.0)	11(64.7)	11(78.6)	0.654^1^^,^^2^
No	9(29.0)	6(35.3)	3(21.4)	
Epilepsy				
Yes	7(22.6)	1(5.9)	6(42.9)	**0.044** ^1^ ^,^ ^2^
No	24(77.4)	16(94.1)	8(57.1)	
Diagnoses				
Vestibular schwannoma WHO I		1		
Well-differentiated chondrosarcoma (G1, focal G2)		1		
Pituitary adenoma		5		
Meningioma WHO I-II		10		
Anaplastic oligodendroglioma WHO III			2	
Anaplastic astrocytoma WHO III			1	
Glioblastoma WHO IV			7	
Hemangiopericytoma WHO III			1	
Metastases			3	

*Group A, benign tumours; Group B, malignant tumours; and SD, standard deviation.*

1 Frequency differences were tested with Chi-squared test;

2 Yates continuity correction was used; and

3 Mean differences were tested with Mann-Whitney U-test.

More women (67.7%) were willing to participate in the study. Mean age, education, relationship status and number of children were similar in the two groups. Epilepsy was found significantly more often in Group B (42.9%) than Group A (5.9%; *χ^2^*(1) = 4.08, *p* = 0.044, *χ* = 0.44; Table 1).

### Distress and Symptom Burden

The analysis of depression demonstrated that the mean value of the total sample (*M* = 9.23, SD = 6.08) did not exceed the cut-off ≥ 10. As depicted in Table 2, more patients in Group B (50.0%) reported symptoms of depression than patients in Group A´(29.4%), but without reaching statistical significance.

**Table 2:** Frequency differences between groups.

		All patients (*N*)	%	Group A (*n*)	%	Group B (*n*)	%	*p* ^1^
PHQ-9	<10	19	61.3	12	70.6	7	50.0	0.423
	≥10	12	38.7	5	29.4	7	50.0	
GAD-7	<10	24	77.4	14	82.4	10	71.4	0.770
	≥10	7	22.6	3	17.6	4	28.6	
PHQ-15	<10	25	80.6	14	82.4	11	78.6	1.000
	≥10	6	19.4	3	17.6	3	21.4	
QSC-R10	<14	15	48.4	8	47.1	7	50.0	1.000
	≥14	16	51.6	9	52.9	7	50.0	
PCL-C	<32	15	82.8	12	70.6	3	21.4	**0.018**
	≥32	16	17.2	5	29.4	11	78.6	
FOP-Q-SF	<50%^2a^	29	93.5	16	94.1	13	92.9	1.000
	≥50%^2b^	2	6.5	1	5.9	1	7.1	

*Group A, benign tumours; Group B, malignant tumours; p, type-I-error probability; PHQ-9, Patient Health Questionnaire; GAD-7, Generalised Anxiety Disorder 7; PHQ-15, 15-question somatic symptom scale from the Patient Health Questionnaire, QSC-R10, Questionnaire on Distress in Cancer Patients-short form; PCL-C, Posttraumatic Stress Disorder Checklist-Civilian Version; FOP-Q-SF, Fear of Progression Questionnaire Short Form.*

1 Frequency differences between Group A and B were tested with chi-squared test and Yates correction for continuity was used. ^2a,b^Percentage of responses with ‘often’ (4 points) or ‘very often’ (5 points).

With regard to anxiety, the entire sample scored below the cut-off value of ≥ 10 (*M* = 6.00, SD = 4.98). 17.6% of the patients in Group A and 28.6% in Group B suffered from symptoms of a generalised anxiety disorder (*p* = 0.770; Table 2).

17.6% of Group A showed a somatic symptom load compared to 21.4% in Group B (medium and high combined ≥ 10; Table 2). These frequency differences did not reach statistical significance.

The sum scores of psychosocial distress were above the cut-off (14 points) in 52.9% of participants in Group A and 50.0% in Group B (*p* = 1.000; Table 2).

With regard to the PCS (physical component summary), Group B reported negligible higher discomfort (*M* = 41.27, SD = 9.60) compared to Group A (*M* = 46.79, SD = 10.14, *p* = 0.142), but these differences did not reach statistical significance. Both groups A and B showed comparable discomfort regarding the MCS (mental component summary; Group A: *M* = 38.91, SD = 12.43 vs. Group B: *M* = 38.38, SD = 11.50, *p* = 0.937; Table 3).

**Table 3:** Distress and symptom burden.

	All patients			Group A			Group B			
	*N*	*M*	SD	*N*	*M*	SD	*N*	*M*	SD	*p* ^1^
PHQ-9	31	9.23	6.08	17	7.88	5.36	14	10.86	6.69	0.297
GAD-7	31	6.00	4.98	17	5.47	5.17	14	6.64	4.58	0.493
PHQ-15	30	5.93	4.84	16	5.31	5.76	14	6.64	3.61	0.131
QSC-R10	30	16.30	11.23	17	14.88	10.56	13	18.15	12.23	0.483
SF-8 PCS	31	44.30	10.13	17	46.79	10.14	14	41.27	9.60	0.142
SF-8 MCS	31	38.67	11.82	17	38.91	12.43	14	38.38	11.50	0.937
PCL-C	31	35.10	13.29	17	32.71	14.99	14	38.00	10.71	0.118
FOP-Q-SF	30	19.33	10.67	16	19.00	11.10	14	19.71	10.56	0.835

*Group A, benign tumours; Group B, malignant tumours; M, mean; SD, standard deviation; p, type-I-error-probability; PHQ-9, Patient Health Questionnaire; GAD-7, Generalised Anxiety Disorder 7; PHQ-15, 15-question somatic symptom scale from the Patient Health Questionnaire; QSC-R10, Questionnaire on Distress in Cancer Patients– short form; SF-8, Short-Form Health Survey; PCS, physical component summary; MCS, mental component summary; PCL-C, Posttraumatic Stress Disorder Checklist-Civilian Version; and FOP-Q-SF, Fear of Progression Questionnaire Short Form.*

1 Mean differences were tested with Mann-Whitney U-test.

The overall posttraumatic stress symptom load was considerably higher in Group B than in Group A (Table 3). 78.6% of the participants in Group B reported clinically relevant posttraumatic stress symptoms (cut-off ≥ 32) compared to 29.4% in Group A. These frequency differences reached statistical significance [*χ^2^*(1) = 5.59, *p* = 0.018, *φ* = 0.49; Table 2].

Only two of the participants (Group A and B) demonstrated fear of progression (Table 2).

## Discussion

We aimed to assess distress in neuro-oncological patients, and in summary, we note that although group differences were not statistical significant in nearly all comparisons, the descriptive results pattern reveals that psychosocial distress was higher in the malignant brain tumour group with regard to symptoms of depression and anxiety, as also post-traumatic stress and somatic symptoms.

We found that the malignant tumour group scored on average above the cut-off value in the PHQ-9 depression module, which strongly suggests major depression symptoms among these patients in contrast to the benign tumour group. Compared to Group B, a more frequent occurrence of depression symptoms in patients with glioma has been described before (Wang et al., 2018; Noll et al., 2019). Noll et al. found depression to be independently associated with shorter overall survival rates in patients with high-grade glioma (Noll et al., 2019). Whether or not psychological intervention can improve the prognosis is still under investigation (Cordier et al., 2019).

Ford et al. reported anxiety in up to 48% of patients with malignant brain tumours (Ford et al., 2012). In our study, we found lower rates of symptoms of anxiety, with 17.6% of the participants in the benign tumour group and 28.6% in the malignant tumour group. The anxiety rates may be underestimated since patients with anxiety were probably less likely to consent to participate in our study. Possibly, due to the existing psychological burden, they may not have sufficient resources to complete several questionnaires addressing anxiety. Moreover, study participation entailed additional conversations, contacts and workload, which anxious patients may have wanted to avoid.

The PHQ-15 demonstrated no group differences in somatic symptoms. In our study, about one-quarter in Group A and Group B scored above the cut-off value of ≥ 10 points. This is of high importance, not only for the patients but also for the caregivers. Somatic symptoms are diverse in neuro-oncological patients and can have a significant impact on daily life.

The physical HRQoL of patients with benign brain tumours (PCS: *M* = 46.79) is comparable with the standard values of healthy peers in Germany (PCS: *M_♀_ =* 45.71, *M_♂_* = 47.46), but participants in the malignant tumour group (PCS: *M* = 41.27) reported lower physical HRQoL. Both groups reported worse mental HRQoL (PCS: benign group *M* = 38.91; malignant group *M* = 38.38) than the healthy and similar-aged population in Germany (MCS: *M_♀_* = 51.06, *M_♂_* = 53.06; Beierlein et al., 2012). This finding underlines that, in addition to medical therapy, psychosocial support is necessary to provide patients with intracranial neoplasia an acceptable level of HRQoL.

Overall, we found higher levels of psychosocial distress in patients with malignant brain tumours. Similarly, this group also demonstrated higher levels of PTSD symptoms. An explanation could be that a diagnosis of brain cancer or metastases provokes death-related distress, given the certainty of tumour progression, lack of curative treatments and poor survival rates (Loughan et al., 2020). It may provoke mortality salience – a traumatic experience – which could be the reason why 79% of the participants with malignant intracranial tumours met the diagnostic criteria for a clinically relevant PTSD and are, therefore, in need of professional psychological treatment. Such distress levels can reduce overall quality of life and therapy adherence, which are critical for a long-lasting positive result, especially in contemporary multimodal cancer treatments. Early detection of distress with immediate intervention is therefore essential [4], especially, because elevated distress levels persist over time and only a fraction of patients with malignant brain tumours receive mental healthcare (Singer et al., 2018). The mean levels of fear of progression were nearly identical in both groups, which does not correspond to the general descriptive results indicating elevated symptom levels in patients with malignant brain tumours. On the one hand, the reason for this may be the time of data collection, since fear of progression occurs more frequently before the start of (radiation-) therapy (Nakata et al., 2020). In this study, however, the data were collected after brain surgery. On the other hand, it has been found in other studies that younger and somatically burdened neuro-oncological patients often report increased fear of progression, which does not apply to our sample (Mehnert et al., 2013; Goebel and Mehdorn, 2019). In addition, it is also conceivable that participants did not yet realise the extent and scope of their therapy and prognosis so soon after diagnosis. In our experience, this mostly happens until the first recurrence or neurological deterioration. Finally, we would also argue that patients with elevated fear of progression are probably less likely to participate in such trials.

### Limitations and Future Directions

Only a fraction of the eligible patients agreed to participate in the study. Therefore, patients with neurological deficits and/or higher levels of psychological distress are probably not adequately represented in our study population. Due to the small sample size, our results may not be estimating the real situation correctly. In addition, the female gender is overrepresented in our sample. Another factor is that, since the surgical methods are more invasive in patients with malignant brain tumours, this cannot be ruled out as a factor leading to the different outcomes between the groups. Although some experience with the instruments implemented in this study exists, neither has been thoroughly validated for neuro-oncologic patients. It turned out that most patients who refused to be enrolled when asked to participate considered the questionnaires to be too long, too time consuming or the questions asked too personal. We also found less variance in the answers on pages five and six, suggesting a loss of motivation. Therefore, we concluded that a comprehensive questionnaire with primarily emotionally loaded items exceeds the capacities of neuro-oncological patients, and it is, therefore, unsuitable.

With regard to appropriate questioning tools, particularly in terms of scope and complexity, there is certainly a need for further research, especially in high-grade gliomas, to optimise recruitment rates and dropout rates in longitudinal studies.

Corresponding efforts have already been made as (i) the DT-BT (distress thermometer brain tumour problem list) is currently being widely validated in multinational samples (Goebel et al., 2020) and (ii) German researchers have developed screening questions for patient-doctor consultation that assess the quality of life and distress of glioma patients, which could prospectively also be implemented in patients with other malignant brain tumours or metastases (Renovanz et al., 2020; Voß et al., 2021).

Furthermore, longitudinal studies – as we initially intended – are important to estimate the long-term development of distress and to identify early risk factors of distress to address the psychosocial concerns of this patient group timeously. Since significant group differences were observed even in our small sample, it is desirable that these findings should be verified in larger samples and, preferably, multicentre studies. Such designs also increase the possibility of generating larger sample sizes, which is challenging due to the rarity of the disease, and could further address insufficient statistical power and recruitment barriers.

### Conclusion

Our study tried to offer a comprehensive assessment of the psychological sequelae of an intracranial tumour diagnosis *via* a combination of standardised questionnaires. We tentatively suggest that, during the course of tumour therapy, psychosocial stress is more often present in patients with malignant intracranial tumours. They also suffer more frequently from epilepsy and PTSD symptoms. A regular screening for distress with instruments adapted to the requirements of neuro-oncological patients should be integrated in the clinical standard procedure and is of the utmost importance to be able to provide psycho-oncological care. Building on that, appropriate interventions have to be developed to address the major goals of patient-centred neuro-oncological therapies: minimising the emotional burden and enabling patients with intracranial tumours to enjoy a maximum degree of quality of life when facing a cancer diagnosis.

## Data Availability Statement

The raw data supporting the conclusions of this article will be made available by the authors, without undue reservation.

## Ethics Statement

The studies involving human participants were reviewed and approved by the Research Ethics Committee of the University of Leipzig, Germany (approval number 467/16-ck). The patients/participants provided their written informed consent to participate in this study.

## Author Contributions

MF, HB, AM-T, and JM contributed to conception and design of the study. MF recruited the patients. MF and HB organized the database. HB and AM-T performed the statistical analysis. MF and HB wrote the first draft of the manuscript. AM-T and JM wrote sections of the manuscript. All authors contributed to manuscript revision, read, and approved the submitted version. MF and HB contributed equally to this work.

## Conflict of Interest

The authors declare that the research was conducted in the absence of any commercial or financial relationships that could be construed as a potential conflict of interest.

## Publisher’s Note

All claims expressed in this article are solely those of the authors and do not necessarily represent those of their affiliated organizations, or those of the publisher, the editors and the reviewers. Any product that may be evaluated in this article, or claim that may be made by its manufacturer, is not guaranteed or endorsed by the publisher.

## Figures and Tables

**Table:** 

Term	Definition
PTSD	posttraumatic stress disorder
CNS	central nervous system
PHQ-9	Patient Health Questionnaire
GAD-7	Generalised Anxiety Disorder 7
PHQ-15	15-question somatic symptoms scale from the Patient Health Questionnaire
QSC-R10	Questionnaire on Distress in Cancer Patients – short form
SF8	Short Form-8 Health Survey
PCL-C	Posttraumatic Stress Disorder Checklist-Civilian Version
FoP-Q-SF	Fear of Progression Questionnaire short form (German: PA-F-KF Kurzform des Progredienzangst-Fragebogens)
HRQoL	Health-related Quality of Life
